# Switching to ziv-aflibercept in resistant diabetic macular edema non responsive to ranibizumab injection

**DOI:** 10.1186/s12886-022-02503-x

**Published:** 2022-06-29

**Authors:** Amin E. Nawar, Tamer Wasfy, Heba M. Shafik

**Affiliations:** Department of Ophthalmology, Faculty of Medicine, Tanta University, Tanta, 31516 Egypt

**Keywords:** Intravitreal injection, Ziv-aflibercept, Optical coherence tomography, Diabetic macular edema

## Abstract

**Background:**

Diabetic macular edema (DME) is a leading cause of visual loss in diabetic patients and is managed using multiple anti-vascular endothelial growth factor (VEGF) agents such as bevacizumab, ranibizumab and aflibercept. The present study evaluates effectiveness of intravitreal injection of ziv-aflibercept in resistant diabetic macular edema.

**Methods:**

This is a prospective interventional study that was carried out on 59 eyes of 40 diabetic patients with diabetic macular edema resistant to three prior consecutive ranibizumab injections. On all patients, thorough ophthalmic evaluation including optical coherence tomography was performed. In patients with persistent intraretinal or subretinal fluid, ziv- aflibercept 1.25 mg (0.05 ml) was administered by intravitreal injection monthly during the 6 month study period from June to December 2019.

**Results:**

The central macular thickness (CMT) decreased significantly from 395.08 ± 129.9 um at baseline to 282.39 ± 95.278, 245.36 ± 79.861 and 201.17 ± 54.042 after 1, 3 and 6 months of treatment respectively (*p* < 0.001). Best corrected visual acuity (BCVA) in log MAR units was significantly improved from 0.95 ± 0.21 to 0.51 ± 0.23 after 6 months (*p* = 0.001). After treatment, negative correlations were detected between age, number of injections, duration of DM and level of glycated hemoglobin (HbA1c) and variation of both CMT and BCVA. The only significant predictor for low final CMT after 6 months of injection was the CMT after 3 months of injection (*p* = 0.001).

**Conclusion:**

Ziv-aflibercept is a highly effective and safe drug in cases of DME resistant to previous ranibizumab injections especially in low-income countries.

**Trial registration:**

This study was retrospectively registered at clinicaltrials.gov (ID: NCT04290195) on 28-2-2020.

## Background

Diabetic macular edema (DME) is a major cause of visual impairment. In the past, DME was managed by focal laser photocoagulation and more recently by intravitreal injections of anti-vascular endothelial growth factor (VEGF) agents and less often by intravitreal dexamethasone implant or intravitreal corticosteroid injections [1].

The safety and efficacy of different anti-VEGF drugs, namely ranibizumab [2] and bevacizumab [3] in the management of DME have been assessed in several studies. The United States Food and Drug Administration (FDA) approved aflibercept to treat DME after the phase 3 trials VIVID and VISTA which provided evidence of significant visual and morphological improvement in patients suffering from DME [4, 5].

According to protocol T of the diabetic retinopathy clinical research network (DRCR.net) [6], aflibercept can be used to treat DME cases, especially those presenting with poor vision. Switching from bevacizumab or ranibizumab to aflibercept is one promising step in managing DME [7–9].

Ziv-aflibercept (Zaltrap; Regeneron, New York, USA), an anti-VEGF drug, is a recombinant fusion protein with a similar mechanism to aflibercept. It was approved by the FDA in August 2012 for the treatment of resistant metastatic colorectal carcinoma. Recently, intravitreal ziv-aflibercept has been considered a safe treatment for age-related macular degeneration, with no ocular toxicity up to 4 weeks after administration [10].

Furthermore, Ziv aflibercept is viewed positively in developing countries as the cost of one dose of intravitreal aflibercept (IVA) and intravitreal ranibizumab (IVR) are $1850 and $1170, respectively, while that for off-label intravitreal bevacizumab (IVB) and intravitreal ziv-aflibercept (IVZ) are $50 and $30 per dose, respectively [11, 12]. Hence, ziv-aflibercept is a safe and inexpensive alternative to other anti VEGF agents for the management of DME.

The present study evaluates the efficacy of ziv-aflibercept (Zaltrap) in cases of resistant diabetic macular edema after previous ranibizumab injections and illustrates the role of proper control of diabetes mellitus (DM) in improving the effectiveness of ziv-aflibercept injection in resistant DME cases.

## Methods

### Study design

A prospective interventional study was conducted on 59 eyes of 40 patients diagnosed with resistant DME after approval from the Ethical Committee of the Faculty of Medicine, Tanta University, Egypt (approval code 32970/02/19). The study cases were recruited in June 2019 and the results were obtained after 6 months in December 2019. All procedures followed the tenets of the Declaration of Helsinki. Written informed consent was given by each participant after discussing the procedure, alternative treatment plans, follow-up schedules and possible benefits and risks.

The study was retrospectively registered with the clinicaltrials.gov database (ID: NCT04290195) on 28-2-2020.

The sample size was calculated using the formula N = (Z 1α + Z1β)^2^(σ1^2^ + σ2^2^)/(m1 − m2)^2^ = 35.

Z1α = 1.96, Z 1β =0.842, σ1, σ2 (standard deviation SD) = (0.33–0.32), m1, m2 = the mean for each group = (0.55–0.33).

### Participants

Patients diagnosed with DME secondary to type 1 or type 2 diabetes mellitus were eligible for inclusion. Those diagnosed with resistance to other treatment were considered for treatment with ziv-aflibercept 1.25 mg/0.05 ml. Patients fulfilling all the following criteria were considered to have resistant DME after at least three consecutive monthly ranibizumab 0.5 mg injections in the previous 6 months: 1-Central macular thickness greater than 300 μm by spectral-domain optical coherence tomography (SD-OCT), 2-Reduction of retinal thickness by less than 10% of baseline retinal thickness, 3-Suboptimal visual improvement (failure to gain at least three lines on the Snellen chart). Thorough ophthalmic evaluation was conducted on all patients, including best corrected visual acuity (BCVA) using Snellen measure converted to log MAR for statistical analysis; intraocular pressure (IOP) measurement using applanation tonometry; anterior segment examination using slit lamp; and posterior segment examination using indirect ophthalmoscopy. Spectral domain optical coherence tomography (SD-OCT) was performed on all patients at presentation and 1 month after the first injection. Patients with history of previous intraocular surgery, coincident retinal pathology such as choroidal neovascular membrane, retinal vein occlusion or age-related macular degeneration, previous laser photocoagulation, or intravitreal injection of triamcinolone acetonide were excluded from the study. Furthermore, patients with prior ocular inflammation, the presence of retinal degeneration and those who did not complete 6 months of follow up were not included in our study.

### Preparation and storage of ziv-aflibercept

Zaltrap (ziv-aflibercept) injection is a clear, colorless to pale-yellow solution supplied in single-dose vials with a concentration of 25 mg/mL. NDC 0024–5840-01: carton containing one single-dose vial of 100 mg/4 mL (25 mg/mL). Zaltrap vials were stored in a refrigerator at 2 °C to 8 °C (36 °F to 46 °F). The vials were kept in the original outer carton to protect from light and the unused portion was discarded. We got the vial from SANOFI medical company after being prescribed by a specialist in the Oncology department in Tanta University for research purpose after being approved by the ethical review board of Tanta University.

### Surgical procedure

The intravitreal injection was carried out in the operating room using a surgical microscope. The eye was prepared using topical anesthesia with one drop of (Benoxinate hydrochloride 0.4% (Benox, Epico, Egypt) to the ocular surface followed by topical instillation of 10% povidone iodine (Betadine) to the eye lashes, lids and periocular area and 5% povidone iodine inside the conjunctival sac 3 min before the procedure. Intravitreal injection of 0.05 ml of 1.25 mg of Ziv-aflibercept (Zaltrap) was administered in the inferotemporal quadrant of the globe using a 30gauge needle 4 mm from the limbus.

### Post-operative care

After the injection, topical antibiotic was administered (Moxifloxacin hydrochloride 0.5% drops, Vigamox, Alcon, USA) with application of an eye patch for several hours.

The patients were examined the next day and the third day after injection to exclude any complications such as increased IOP, endophthalmitis, retinal break, retinal detachment or vitreous hemorrhage. All patients were followed up at 4-week intervals after the first injection for 6 month duration of the study. At each visit a thorough ophthalmic examination was performed including BCVA and SD-OCT. Additional intravitreal injection of Zaltrap was given after 1 month if persistent intraretinal or subretinal fluid was detected on SD-OCT.

### Statistical analysis

Statistical presentation and analysis of the data were conducted using the mean, standard deviation, Student’s t- test, Chi-square, Linear Correlation Coefficient and analysis of variance [ANOVA] tests in the Statistical Package for the Social Sciences (SPSS, Chicago, IL, USA). Unpaired Student’s t-test was used to compare between two groups in quantitative data. Chi-square indicates that the row and column variables are independent, without indicating strength or direction of the relationship. Linear correlation analysis was used to look for correlations between two quantitative variables in one group. Analysis of variance [ANOVA] test was used for comparison between quantitative data collected at the different time intervals in the same group. Multivariate regression analyses were used to assess predictors of final anatomical results with involvement of significant predictors only. *P*-values ≤0.05 were considered significant.

## Results

**Fig. 1:**
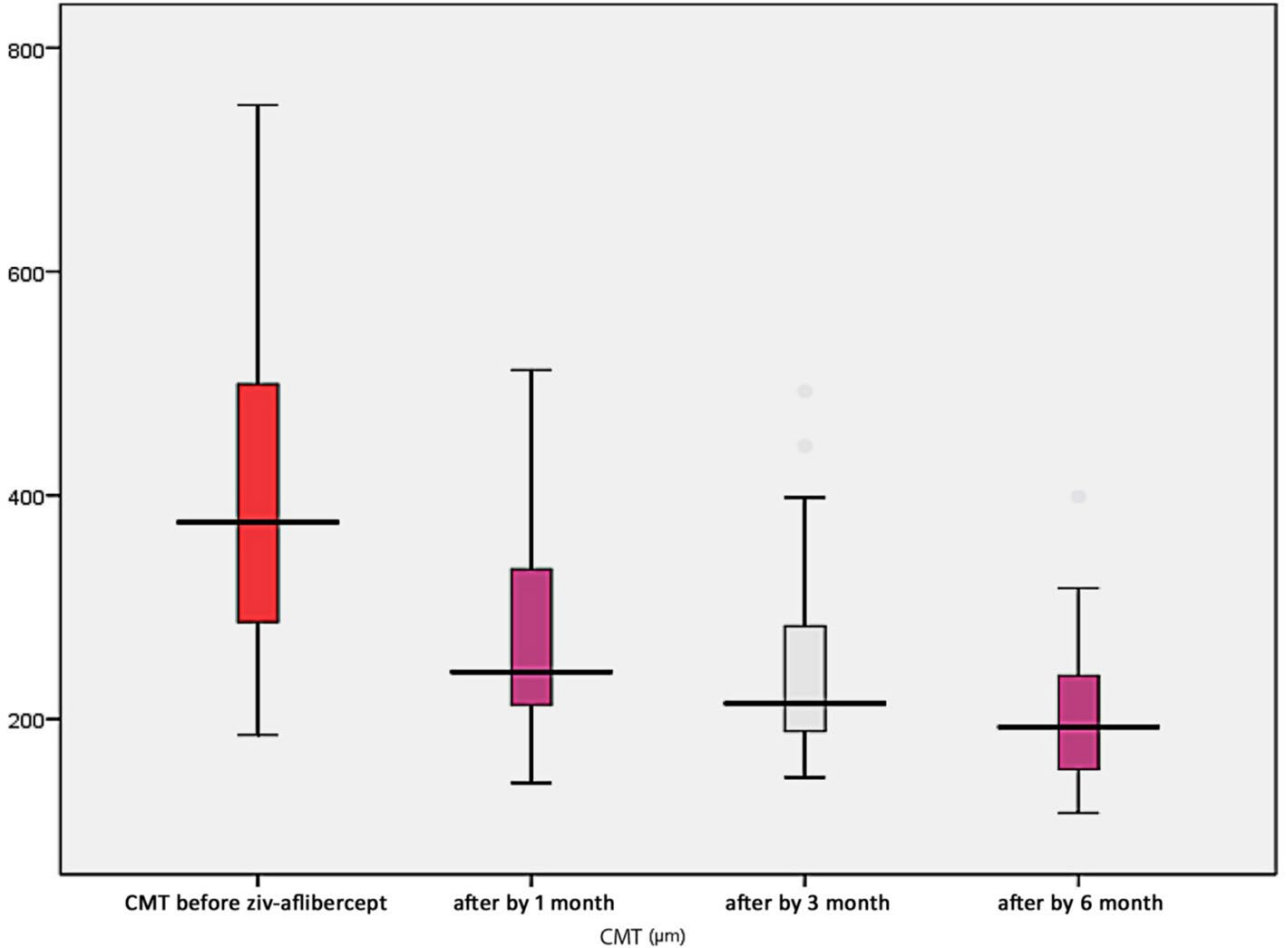
Variation in mean central macular thickness (um) before and after ziv-aflibercept injection

**Fig. 2:**
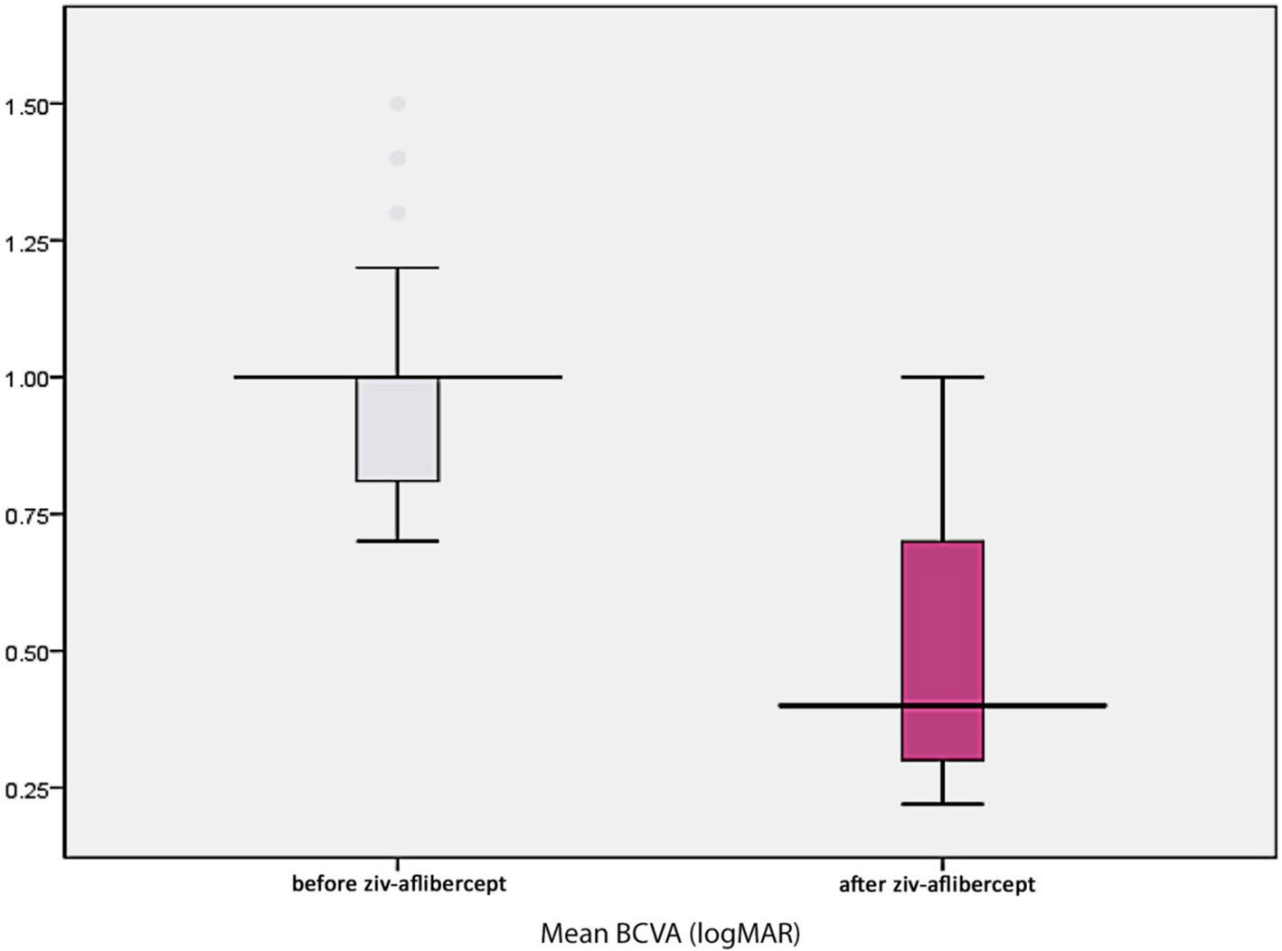
Variation in mean BCVA by logMAR before and after ziv-aflibercept injections

The baseline demographic and clinical data of all patients are shown in Table 1. The mean age of patients was 51.36 ± 6.98; 22 females and 18 males were included in the study. Sixteen patients were under insulin treatment and the remaining 24 patients were receiving oral treatment. The mean number of intravitreal injections of ziv-aflibercept patients had received throughout the study was 3.52 ± 1.4 and the mean level of HbA1c was 8.76 ± 0.99. All patients were referred to the internal medicine department for proper control of HbA1c throughout the duration of the study. The central macular thickness (CMT) was significantly decreased from 395.08 ± 129.9 um at baseline to 282.39 ± 95.278, 245.36 ± 79.861 and 201.17 ± 54.042 after 1, 3 and 6 months of injection respectively (*p* < 0.001; Table 2, Fig. 1). The log MAR BCVA improved from 0.95 ± 0.21 to 0.51 ± 0.23 after the six-month injection (*p* = 0.001; Table 1, Fig. 2). Table 3 shows correlations between different predictors of response to ziv-aflibercept injection with variation in CMT and BCVA after the switch to ziv-aflibercept. Significant negative correlations were found between CMT variation and the following three factors: number of injections, duration of DM and HbA1c level (*p* < 0.003). Correlations between these factors and BCVA were not significant, however (*p* > 0.05). Results of multivariate linear regression analyses are presented in Table 4, and show that the CMT after 3 months was the only significant predictor for final CMT at 6 months (*p* = 0.001).

**Table 1:** Demographics and clinical characteristics of patients

Age (years)	
Mean ± SD	51.36 ± 6.98
Sex	
Female (n, % of total)	22 (55%)
Male (n,% of total)	18 (45%)
Type of ttt	
Insulin (n, % of total)	16 (40%)
Oral(n,% of total)	24 (60%)
No of injections (mean ± SD)	3.52 ± 1.4
BCVA before injection (mean ± D)	0.95 ± 0.21
BCVA after 6 months	
mean ± SD	0.51 ± 0.23
Median (IQR)	0.4 (0.3–0.7)
Duration of DM Median (IQR)	10 (7–15)
HbA1c	8.76 ± 0.99

*n* Number, *BCVA* Best corrected visual acuity, *SD* Standard deviation, *DM* Diabetes mellitus, *IQR* Interquartile range, *HbA1c* Hemoglobin A1c

**Table 2:** Central macular thickness (CMT) before injection and after one, three and six months of injection

	Before injection	After 1 m.	After 3 m.	After 6 m.	
	395.08 ± 129.9um	282.39 ± 95.278um	245.36 ± 79.861um	201.17 ± 54.042um	
**F. test**	46.09				
***P*. value**	< 0.001				
Before injection After 1 m.	Before injection and After 3 m.	Before injection and After 6 m	After 1 m &3 month	After 1 m &6 month	After 3 m &6 month
5.4 0.01*	7.6 < 0.001*	10.5 < 0.001*	2.3 0.02*	5.6 < 0.001*	3.4 0.001*

*CMT* Central macular thickness, *statistically significant

**Table 3:** Correlations between possible predictors for response to ziv-aflibercept

			Δ CMT	Δ BCVA
Spearman’s rho	Age	R	−0.253	−0.146
		*P* value	0.053	0.270
	No of injections	R	−0.435	− 0.183
		*P* value	0.001^*^	0.166
	Duration of DM	R	−0.390	−0.165
		*P* value	0.002^*^	0.211
	HbA1c	R	−0.510	−0.023
		*P* value	0.001^*^	0.862

*CMT* Central macular thickness, *BCVA* Best corrected visual acuity, *HbA1c* Hemoglobin A1c, *statistically significant, *DM* Diabetes mellitus, Presented values correspond to the linear correlation coefficient (R). Δ Visual acuity: variation of best corrected visual acuity after ziv-aflibercept. Δ Macular thickness: variation of central macular thickness after ziv-aflibercept

**Table 4:** Multivariate linear regression analysis for predictors of ziv-aflibercept anatomical response after 6 months

			*P* value	95% Confidence Interval for B	
	B	Beta		Lower Bound	Upper Bound
No of injection	2.034	0.054	0.667	−7.404	11.471
BCVA before injection	−24.807	−0.097	0.471	−93.435	43.822
Final BCVA after 6 months	1.694	0.008	0.947	− 49.466	52.855
Duration	1.335	0.129	0.237	−0.906	3.577
HbA1c	−3.894	−0.071	0.536	−16.437	8.648
pre switch CMT	−0.007	− 0.018	0.911	− 0.140	0.125
CMT after one month	−0.107	− 0.188	0.291	−0.307	0.094
CMT after three month	0.645	0.953	0.001^*^	0.423	0.866

*No* Number, *BCVA* Best corrected visual acuity, *CMT* Central macular thickness, *HbA1c* Hemoglobin A1c, *statistically significant

Subconjunctival hemorrhage occurred in five eyes after injection and resolved spontaneously. No major ocular complications such as glaucoma, ocular hypertension, endophthalmitis, vitreous hemorrhage, or retinal detachment occurred. No serious systemic complications such as stroke, myocardial infarction or death were reported during the follow up period of the study.

**Fig. 3:**
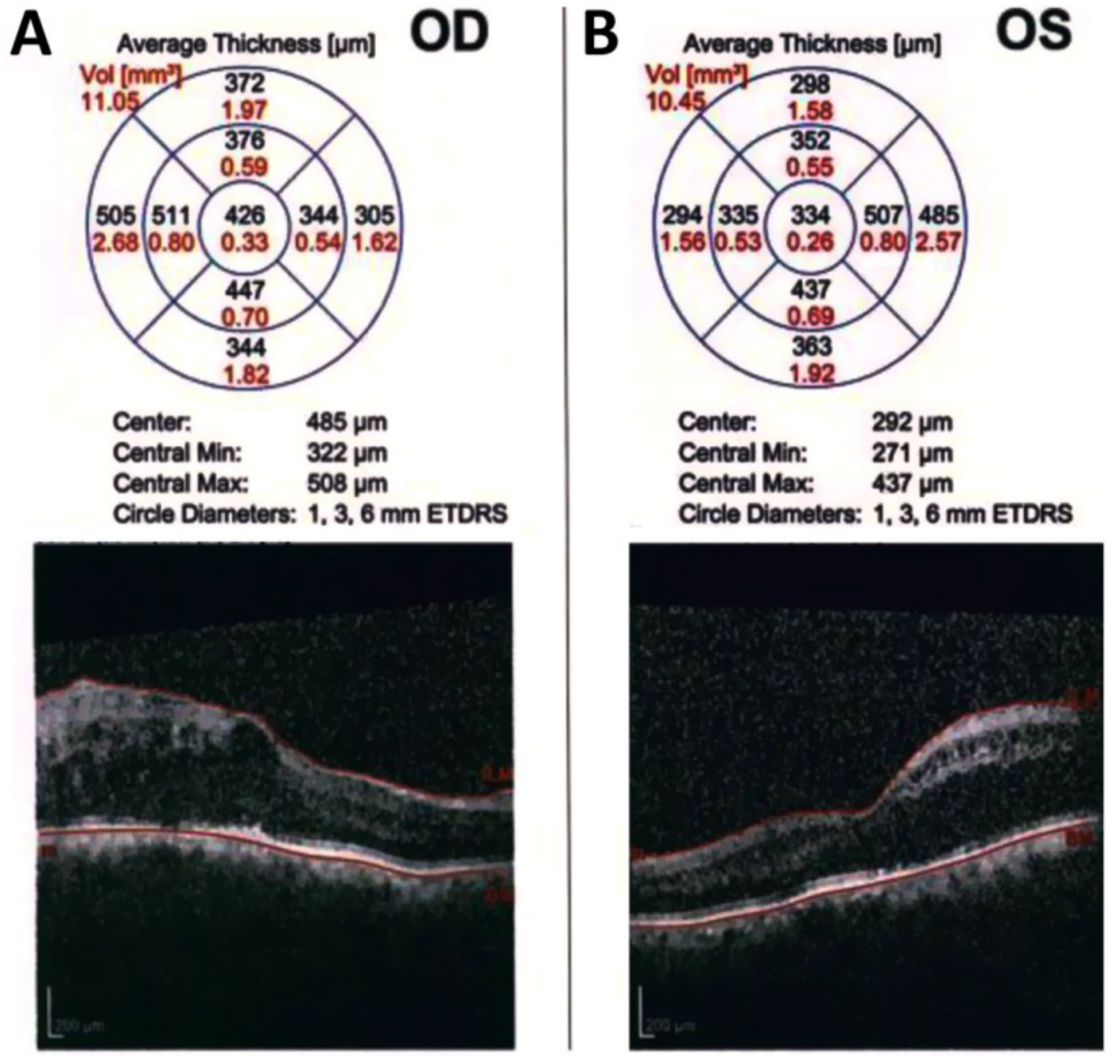
A case of bilateral diabetic macular edema with CMT 426 um in the right eye (**A**), and 334 um in the left eye (**B**). The BCVA is 1(log MAR) in the right eye and 0.82 (log MAR) in the left eye

**Fig. 4:**
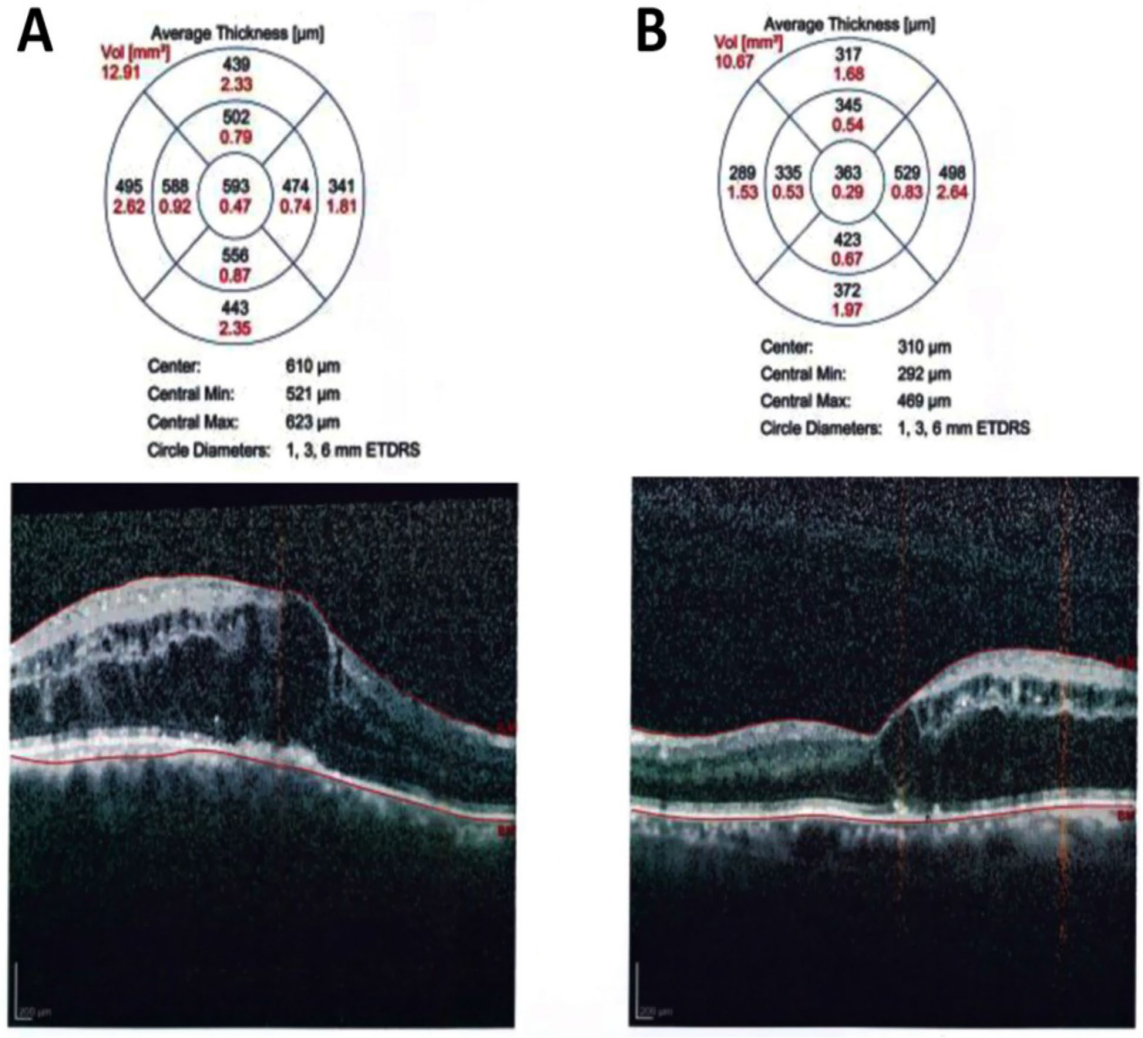
OCT of the case shown in Fig. 3 after 3 loading doses of ranibizumab in each eye. The CMT did not decrease after injection, the CMT is 593 um in the right eye (**A**) and 363 um in the left eye (**B**); the BCVA did not improve after injection, the BCVA is 1 (log MAR) in the right eye and 0.82 (log MAR) in the left eye

**Fig. 5:**
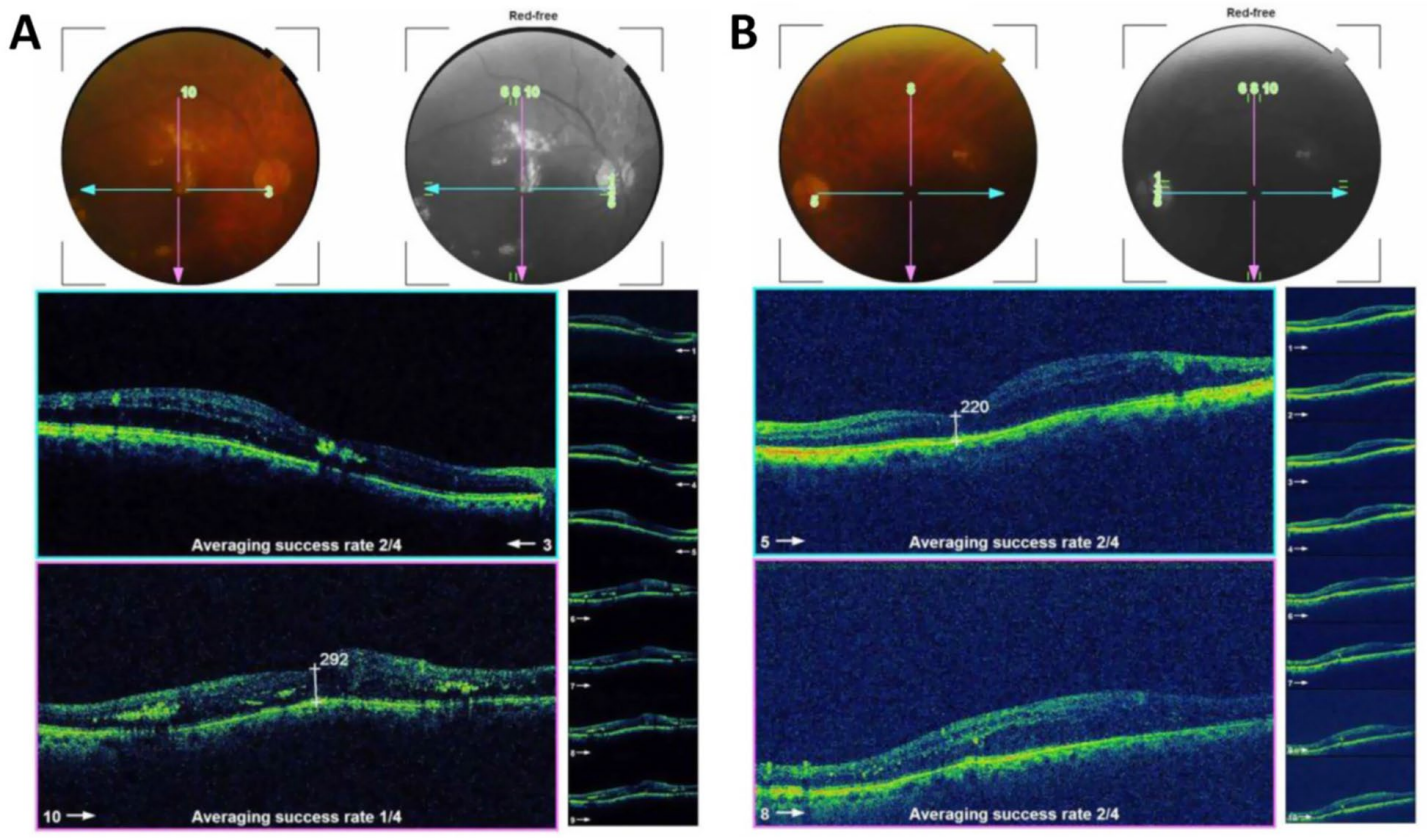
OCT of the case shown in Fig. 3 after one month of the first injection of ziv-aflibercept. The CMT decreased to 292 um in the right eye (**A**) and 220 um in the left eye (**B**)

**Fig. 6:**
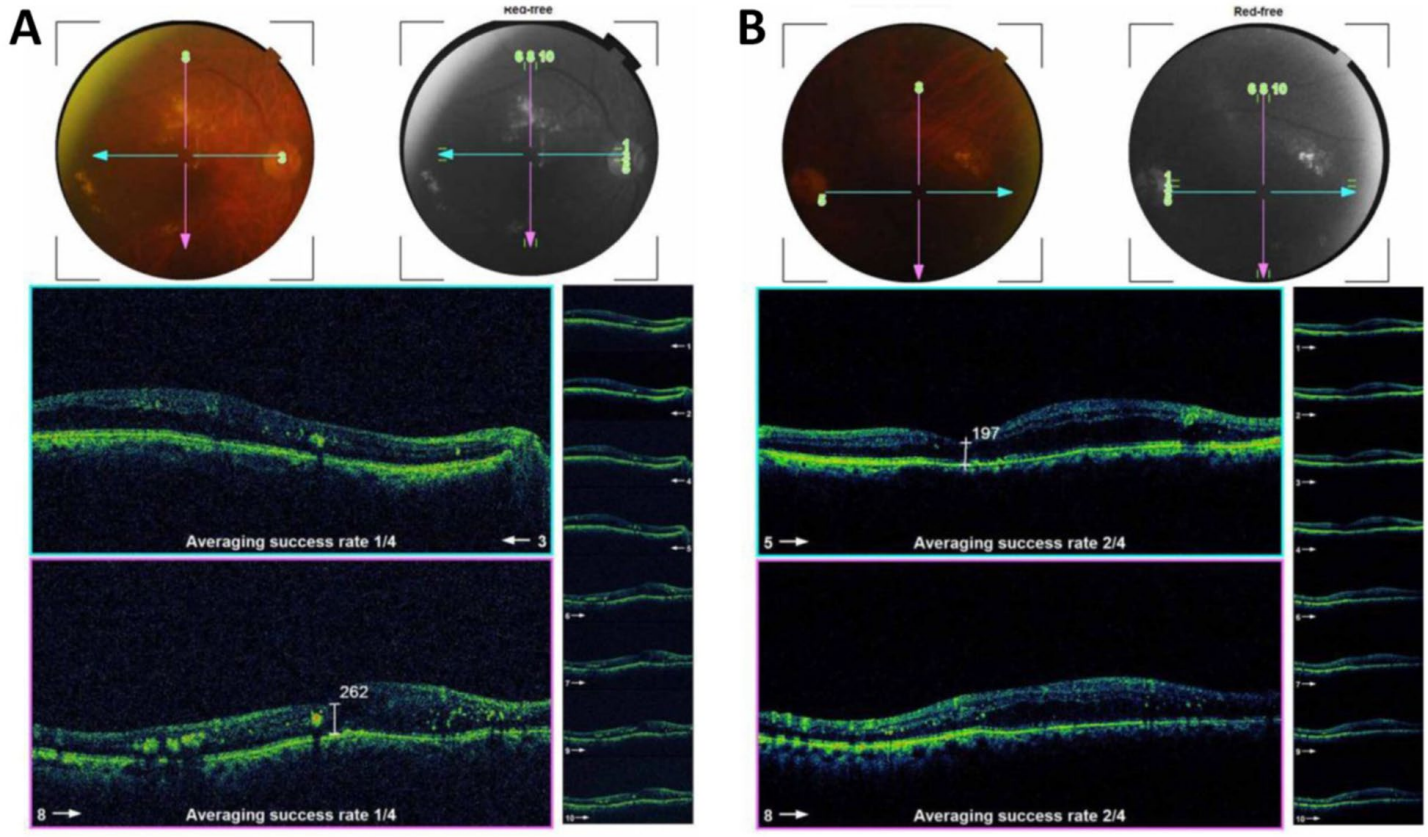
OCT of the case shown in Fig. 3 after 3 injections of ziv-aflibercept (after 3 months). The CMT decreased to 262 um in the right eye (**A**) and 197 um in the left eye (**B**)

**Fig. 7:**
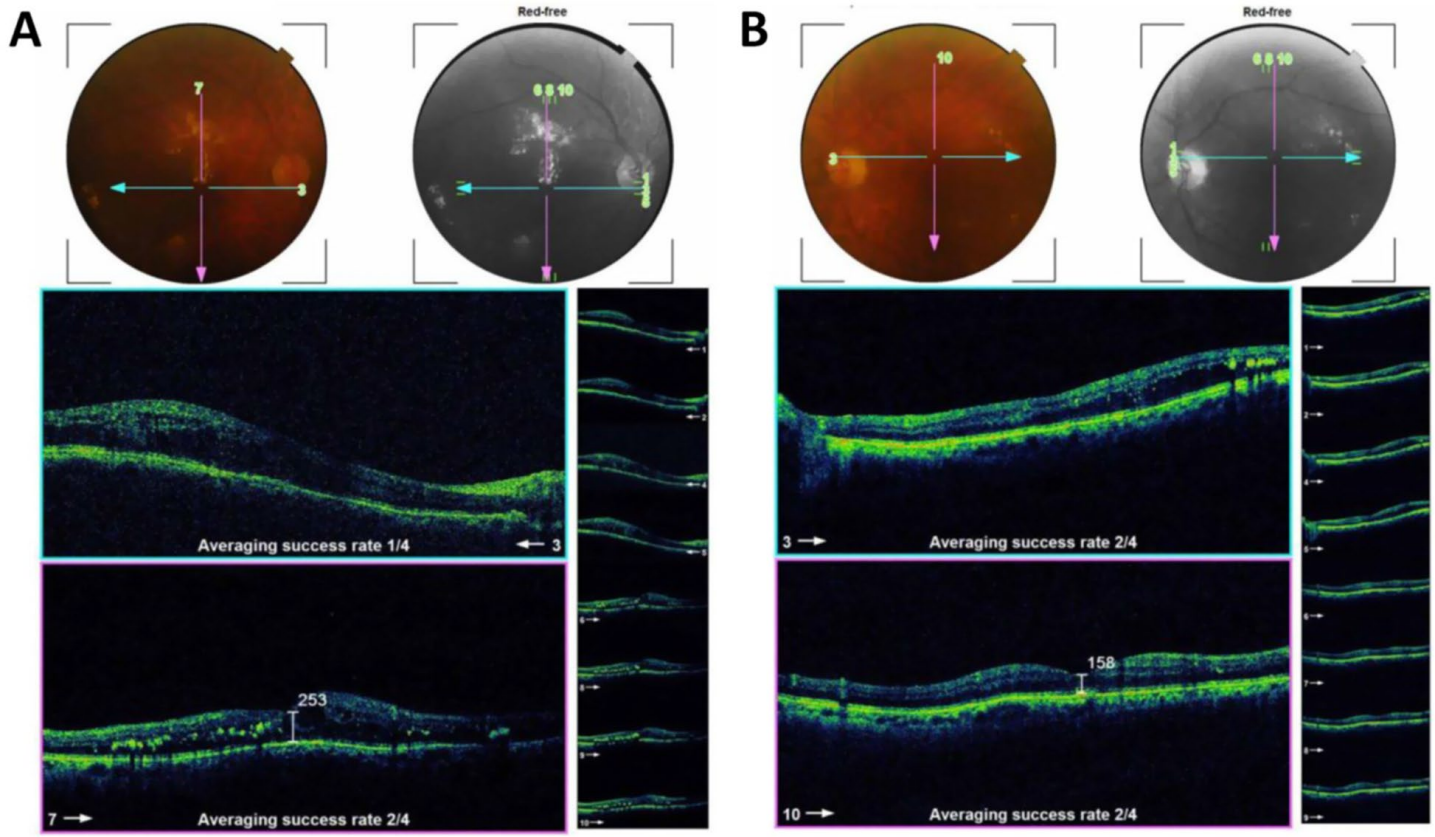
OCT of the case shown in Fig. 3 after 6 ziv-aflibercept injections (after 6 months) showing decreased CMT to 253 um in the right eye (**A**) and 158 um in the left eye (**B**); the BCVA improved to 0.3 (log MAR) in the right eye and 0.4 (log MAR) in the left eye

Figure 3 is an example of a case of bilateral diabetic macular edema with CMT 426 um in the right eye (Fig. 3A), and 334 um in the left eye (Fig. 3B). The BCVA is 1(log MAR) in the right eye and 0.82 (log MAR) in the left eye. Figure 4: OCT of the case shown in Fig. 3 after 3 loading doses of ranibizumab in each eye. The CMT did not decrease after injection, the CMT is 593 um in the right eye (Fig. 4A) and 363 um in the left eye (Fig. 4B); the BCVA did not improve after injection, the BCVA is 1 (log MAR) in the right eye and 0.82 (log MAR) in the left eye. Figure 5: OCT of the case shown in Fig. 3 after 1 month of the first injection of ziv-aflibercept. The CMT decreased to 292 um in the right eye (Fig. 5A) and 220 um in the left eye (Fig. 5B), Fig. 6: OCT of the case shown in Fig. 3 after 3 injections of ziv-aflibercept (after 3 months). The CMT decreased to 262 um in the right eye (Fig. 6A) and 197 um in the left eye (Fig. 6B). Figure 7: OCT of the case shown in Fig. 3 after 6 ziv-aflibercept injections (after 6 months) showing decreased CMT to 253 um in the right eye (Fig. 7A) and 158 um in the left eye (Fig. 7B); the BCVA improved to 0.3 (log MAR) in the right eye and 0.4 (log MAR) in the left eye.

## Discussion

DME is mainly caused by increased retinal vascular permeability leading to the accumulation of fluid in the retina with subsequent increase in its thickness. These events are associated with disruption of the blood retinal barrier and increased production of VEGF [13, 14]. Several randomized clinical trials have demonstrated the efficacy of VEGF inhibitors (anti-VEGF) in the treatment of DME with improvement in visual acuity and a reduction in central macular thickness (CMT) [1, 2, 4, 5].

Intravitreal injection of ziv-aflibercept has been used in multiple chorioretinal conditions with great success especially in the developing countries [15–18]. In the present study, we investigated the safety and the efficacy of intravitreal injection of 1.25 mg of ziv-aflibercept in 59 eyes with diabetic macular edema resistant to previous ranibizumab injection.

No cases of uveitis or endophthalmitis were reported in our study indicating high safety profile of the new drug. However, a previous study reported 0.03–0.05% incidence of endophthalmitis after injection of bevacizumab, ranibizumab and aflibercept [18].

Among the 59 eyes that received intravitreal injection of ziv-aflibercept in the present study, no cases of glaucoma or ocular hypertension were reported. In contrast, previous studies have reported increased IOP in 6.25–33% of eyes with glaucoma or ocular hypertension and in 1.6–7.1% of non-glaucomatous eyes following anti-VEGF injections [19–21]. The present study found marked functional and anatomical improvements after 6 months of follow up, with significant improvement in BCVA and marked reduction in CMT.

In agreement with our study, two randomized controlled trials have found the new drug to be effective. Intravitreal ziv-aflibercept (IVZ) and intravitreal bevacizumab (IVB) treatments were compared in 123 eyes with DME. Patients were randomized to one of three loading doses of 1.25 mg IVZ, 2.5 mg IVZ or 1.25 mg IVB administered by injection. At 12 weeks both doses of ziv-aflibercept achieved similar results with greater visual improvement than IVB [16]. After 12 weeks, IVB was injected every 4 weeks, whereas both IVZ groups were injected every 8 weeks through 1 year, after which BCVA outcomes were better in patients with IVZ than in those with IVB treatment [22].

Other studies have confirmed the efficacy of ziv-aflibercept, in support of our study. The earliest of these was performed on 50 eyes, with ziv-aflibercept injections in 27 eyes and bevacizumab was injected in 23 eyes in a pro-re-nata regimen (PRN). Both groups achieved similar improvements in mean visual acuity at 3 months but patients receiving ziv-aflibercept required fewer injections (2.4 vs. 3.6) [23, 24].

Another 30-month prospective study assessed the efficacy of ziv-aflibercept in18 eyes with DME in a treat and extend regimen. At the 30-month examination, improvements in both mean CMT (*p* = 0.027) and mean visual acuity (*p* = 0.042) were reported [25].

In this study, we aimed to investigate the role of proper control of DM and its duration on the anatomical and functional response after a switch to ziv-aflibercept treatment. Our correlation analysis findings indicate poorer response to ziv-aflibercept in patients with poorer metabolic control, higher HbA1c or longer duration of DM. This is not in agreement with a previous study on treatment switching from bevacizumab to aflibercept, which found no association between control or duration of DM and response to aflibercept [26].

Our investigation of the possible predictors for better anatomical response to ziv-aflibercept found that the only significant predictor for this outcome was the central macular thickness 3 months after treatment onset. Thus, cases with lower CMT after 3 months showed better anatomical response after 6 months of ziv-aflibercept injection. In contrast, one other study reported that the baseline CMT before the treatment switch is the only significant predictor for a better anatomical response after switching to aflibercept [26]. The cost of the treatment procedure in the present study included the hospital costs, hospital user fees, cost of investigations such as OCT, fluorescein angiography, and cost of the anti-VEGF drug. The mean number of injections was 3.52 ± 1.4. The costs of one dose of IVB and IVZ are similar ($50 and $30 per dose, respectively) [11, 17]. However, the cost of IVA or IVR is 20–30 times this amount [27]. The relative affordability of IVB or IVZ may be of great benefit to the patients in the developing and low-middle-income countries where there is limited insurance coverage. In addition, a reduction in the number of hospital visits required for patients who receive IVZ may reduce further costs.

A limitation of this study is the small sample with short duration of follow up. In further research, a larger number of patients needs to be evaluated with a longer follow up period to assess the efficacy of this new drug.

## Conclusion

The study confirmed the efficacy, safety and the cost effectiveness of ziv-aflibercept in cases of diabetic macular edema resistant to prior ranibizumab injections. In addition, the worse DM control and the higher baseline HbA1C level, the less response to ziv-aflibercept injection. In developing countries ziv-aflibercept may be used to replace other more expensive agents.

## Data Availability

The datasets used during the current study are available from the corresponding author on reasonable request.

## Abbreviations

ANOVA: Analysis of variance
BCVA: Best corrected visual acuity
CMT: Central macular thickness
DME: Diabetic macular edema
DRCR.net: Diabetic retinopathy clinical research network
FDA: Food and drug administration
FFA: Fundus fluorescein angiography
HbA1c: Hemoglobin A1c
IOP: Intraocular pressure
IVA: Intravitreal aflibercept
IVB: Intravitreal bevacizumab
IVR: Intravitreal ranibizumab
IVZ: Intravitreal ziv-aflibercept
OCT: Optical coherence tomography
PRN: Pro-re-nata
SPSS: Statistical package for the social sciences
VEGF: Vascular endothelial growth factor

## References

[1] Mitchell P, Bandello F, Schmidt-Erfurth U, Lang GE, Massin P, Schlingemann RO (2011). The RESTORE study: ranibizumab monotherapy or combined with laser versus laser monotherapy for diabetic macular edema. Ophthalmology.

[2] Nguyen QD, Brown DM, Marcus DM, Boyer D, Patel S, Feiner L (2012). Ranibizumab for diabetic macular edema: results from 2 phase III randomized trials: RISE and RIDE. Ophthalmology.

[3] Rajendram R, Fraser-Bell S, Kaines A, Michaelides M, Hamilton RD, Degli Esposti S (2012). A 2-year prospective randomized controlled trial of intravitreal bevacizumab or laser therapy (BOLT) in the management of diabetic macular edema: 24-month data: report 3. Arch Ophthalmol.

[4] Brown DM, Schmidt-Erfurth U, Do DV, Holz FG, Boyer DS, Midena E (2015). Intravitreal aflibercept for diabetic macular edema: 100-week results from the VISTA and VIVID studies. Ophthalmology.

[5] Do DV, Nguyen QD, Boyer D, Schmidt-Erfurth U, Brown DM, Vitti R (2012). One-year outcomes of the da Vinci study of VEGF trap-eye in eyes with diabetic macular edema. Ophthalmology.

[6] Wells JA, Glassman AR, Jampol LM, Aiello LP, Antoszyk AN, Diabetic Retinopathy Clinical Research Network (2015). Afliibercept, bevacizumab, or ranibizumab for diabetic macular edema. N Engl J Med.

[7] Ashraf M, Souka AA, ElKayal H (2017). Short-term effects of early switching to ranibizumab or aflibercept in diabetic macular edema cases with non-response to bevacizumab. Ophthalmic Surg Lasers Imaging Retina.

[8] Bahrami B, Hong T, Zhu M, Schlub TE, Chang A (2017). Switching therapy from bevacizumab to aflibercept for the management of persistent diabetic macular edema. Graefes Arch Clin Exp Ophthalmol.

[9] Chen YY, Chang PY, Wang JK (2017). Intravitreal aflibercept for patients with diabetic macular edema refractory to bevacizumab or ranibizumab: analysis of response to aflibercept. Asia Pac J Ophthalmol (Phila).

[10] Chhablani J, Narayanan R, Mathai A, Yogi R, Stewart M (2016). Short-term safety profile of intravitreal ziv-aflibercept. Retina.

[11] Mansour AM, Al-Ghadban SI, Yunis MH, El-Sabban ME (2015). Ziv-aflibercept in macular disease. Br J Ophthalmol.

[12] Mansour AM, Ashraf M, Dedhia CJ, Charbaji A, Souka AA, Chhablani J (2017). Long-term safety and efficacy of ziv-aflibercept in retinal diseases. Br J Ophthalmol.

[13] Polo RC, Sánchez CR, Guisado DMG, Luque MJD (2018). Aflibercept for clinically significant diabetic macular edema: 12-month results in daily clinical practice. Clin Ophthalmol.

[14] Fouda SM, Bahgat AM (2017). Intravitreal aflibercept versus intravitreal ranibizumab for the treatment of diabetic macular edema. Clin Ophthalmol.

[15] Mansour AM, Ashraf M, Charbaji A, Younis MH, Souka AA, Dogra A (2018). Two-year out- comes of intravitreal ziv-aflibercept. Br J Ophthalmol.

[16] Baghi A, Bonyadi MHJ, Ramezani A, Azarmina M, Moradian S, Dehghan MH (2017). Two doses of intravitreal ziv-aflibercept versus bevacizumab in treatment of diabetic macular edema: a three-armed, double-blind randomized trial. Ophthalmol Retina.

[17] Singh SR, Dogra A, Stewart M, Das T, Chhablani J (2017). Intravitreal ziv-aflibercept: clinical effects and economic impact. Asia Pac J Ophthalmol (Phila)..

[18] Singh SR, Stewart MW, Chattannavar G, Ashraf M, Souka A, ElDardeery M (2019). Safety of 5914 intravitreal ziv-aflibercept injections. Br J Ophthalmol.

[19] Good TJ, Kimura AE, Mandava N, Kahook MY (2011). Sustained elevation of intraocular pressure after intravitreal injections of anti-VEGF agents. Br J Ophthalmol.

[20] Hoang QV, Tsuang AJ, Gelman R, Mendonca LS, Della Torre KE, Jung JJ (2013). Clinical predictors of sustained intraocular pressure elevation due to intravitreal anti-vascular endothelial growth factor therapy. Retina.

[21] Wehrli SJ, Tawse K, Levin MH, Zaidi A, Pistilli M, Brucker AJ (2012). A lack of delayed intraocular pressure elevation in patients treated with intra- vitreal injection of bevacizumab and ranibizumab. Retina.

[22] Jabbarpoor Bonyadi MH, Baghi A, Ramezani A, Yaseri M, Soheilian M (2018). One-year results of a trial comparing 2 doses of intravitreal ziv-aflibercept versus bevacizumab for treatment of diabetic macular edema. Ophthalmol Retina.

[23] Ashraf M, El Kayal H, Souka AAR (2017). Comparison between the short-term outcomes of bevacizumab and ziv-aflibercept in the treatment of primary diabetic macular oedema. Acta Ophthalmol.

[24] Ashraf M, Kayal HE, Souka AAR (2017). Safety and efficacy of ziv-aflibercept in the treatment of refractory diabetic macular edema. Ophthalmic Surg Lasers Imaging Retina.

[25] Mansour AM, Charbaji A, Farah ME, Mansour HA, Chhablani J (2019). Long-term out- come of treat and extend intravitreal ziv-aflibercept therapy. Br J Ophthalmol.

[26] Laiginhas R, Silva MI, Rosas V, Penas S, Fernandes VA, Rocha-Sousa A (2018). Aflibercept in diabetic macular edema refractory to previous bevacizumab: outcomes and predictors of success. Graefes Arch Clin Exp Ophthalmol.

[27] Ross EL, Hutton DW, Stein JD, Bressler NM, Jampol LM, Glassman AR (2016). Cost-effectiveness of aflibercept, bevacizumab, and ranibizumab for diabetic macular edema treatment: analysis from the diabetic retinopathy clinical research network comparative effectiveness trial. JAMA Ophthalmol.

